# Laser-Assisted Surgical Management of Oral Mucocele: A Case Report

**DOI:** 10.7759/cureus.53020

**Published:** 2024-01-26

**Authors:** Sourabh Shinde, Vidya Lohe, Unnati Shirbhate, Swapnil Mohod, Dhruvi Solanki

**Affiliations:** 1 Oral Medicine and Radiology, Sharad Pawar Dental College, Datta Meghe Institute of Higher Education and Research, Wardha, IND; 2 Periodontics, Sharad Pawar Dental College, Datta Meghe Institute of Higher Education and Research, Wardha, IND; 3 Pedodontics and Preventive Dentistry, Sharad Pawar Dental College, Datta Meghe Institute of Higher Education and Research, Wardha, IND

**Keywords:** histopath, diode laser, retention, extravasation phenonmenon, mucocele

## Abstract

This case represents mucocele of extravasation phenomenon associated with a lower lip on the right side in the last 15 days. A 19-year-old male patient visited the outpatient department with a history of constant trauma due to lip biting and due to soft and flocculent consistency on palpation, mucocele was considered under the provisional diagnosis. The borders of the lesion were marked following all the protocols of asepsis and sterilization and laser-assisted surgical excision was undertaken resulting in total removal of the lesion with a diode laser by resecting it from the base to reduce chances of re-occurrence. The specimen that was resected was sent for histopathological examination, which confirmed the final diagnosis of mucous extravasation cyst or mucocele. The following report underlines that laser-assisted resection offers a minimally invasive and precision approach for the treatment of mucocele.

## Introduction

A common oral mucosal lesion, mucocele is a result of mucous accumulation that occurs because of minor salivary gland alteration [1-4]. Its occurrence can be seen anywhere there is the presence of minor salivary glands and is classified into two types- extravasation and retention. The lower lip is the most common site for extravasation while there is no common site for retention type [1,2]. It presents as restricted swelling caused by accumulation of mucin which is bluish, soft, transparent cystic swelling that usually resolves on its own. Whenever the duct ruptures and there is a spillage around the gland, it results in an extravasation type of cyst, but when there is a blockage of the duct diminishing the discharge, a retention type of cyst results. When the floor of the mouth is involved and resembles the belly of a frog it is called a Ranula. These lack epithelial lining and may also termed superficial mucocele and classical mucocele [3,4]. The one that affects the mucous membrane is the superficial mucocele whereas the one that affects the submucosa is the classical mucocele [1-4].

Mucoceles present on labial mucosa, buccal mucosa, and retromolar area usually do not have the epithelial lining [5,6]. They trigger secondary inflammation which results in the periodic discharge of mucous from the concerned area, about which the patient frequently complains. The retention type, which is seen less frequently than the extravasation type, is usually present on the hard palate, floor of the mouth, upper lip, and maxillary sinus region. The retention of the mucous is due to obstruction of the duct by either sialolith or strictures. This can also be due to ductal narrowing which can be caused by deodorant mouthwashes, tartar control toothpaste, hydrogen peroxide, or antiplaque solution which irritates the mucosa [7,8]. The major factor that is involved in etiopathogenesis is obstruction as a result of trauma involving the duct of the involved salivary gland. As discussed earlier, the spillage of salivary secretions is a result of traumatic injuries to the gland duct and is the cause of the extravasation type. This is followed by the stagnation of the mucous that results in inflammation, which can be aggravated by habits like lip biting and tongue thrusting. The extravasation type undergoes three different phases: in Phase I after the spillage there is the presence of leukocytes and histiocytes [9,10]; in Phase II there is the presence of granulomas due to the presence of macrophages and histiocytes. There is also the presence of multinucleated giant cells that are due to foreign body reactions. It is also termed as the resorption phase. Phase III involves the presence of a pseudo capsule that shows no epithelium surrounding the mucosa because of connective tissue cells. Retention type is a feature commonly linked to major salivary glands, occurring by the blockage of the duct due to sialolith or dense mucosa. The obstruction is usually from the secretory apparatus of the gland [10,11].

## Case presentation

**Figure 1:**
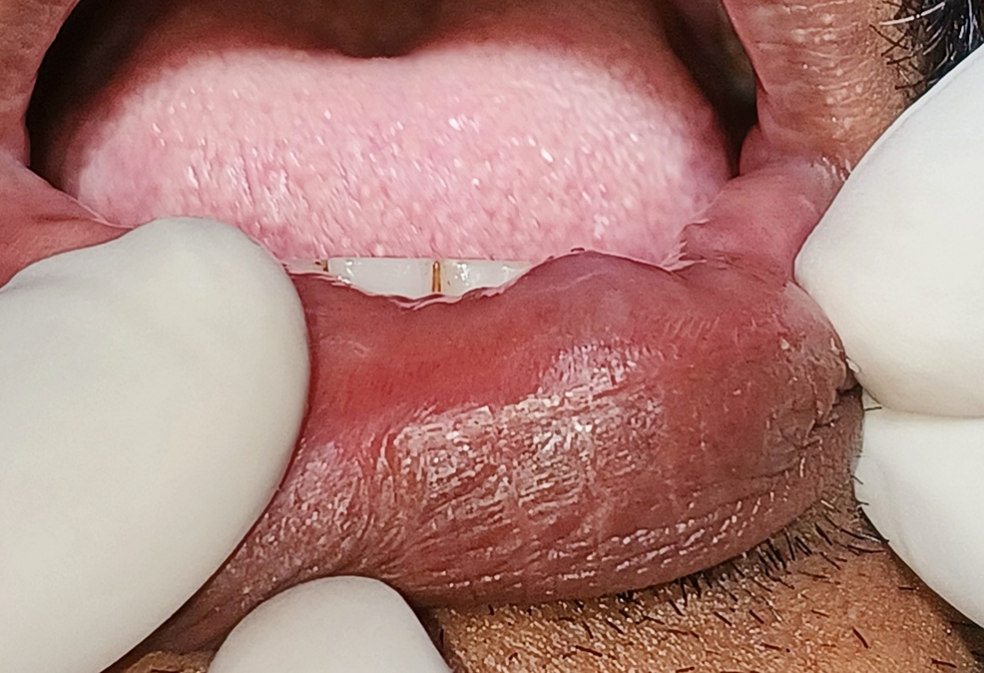
Pre-operative view of mucous extravasation cyst associated with lower lip.

A 19-year-old male patient visited the oral physician in the outpatient department complaining of painless swelling of the lower lip on the right side since 15 days. The patient presented with a history of constant trauma due to lip biting as shown in Figure 1.

**Figure 2:**
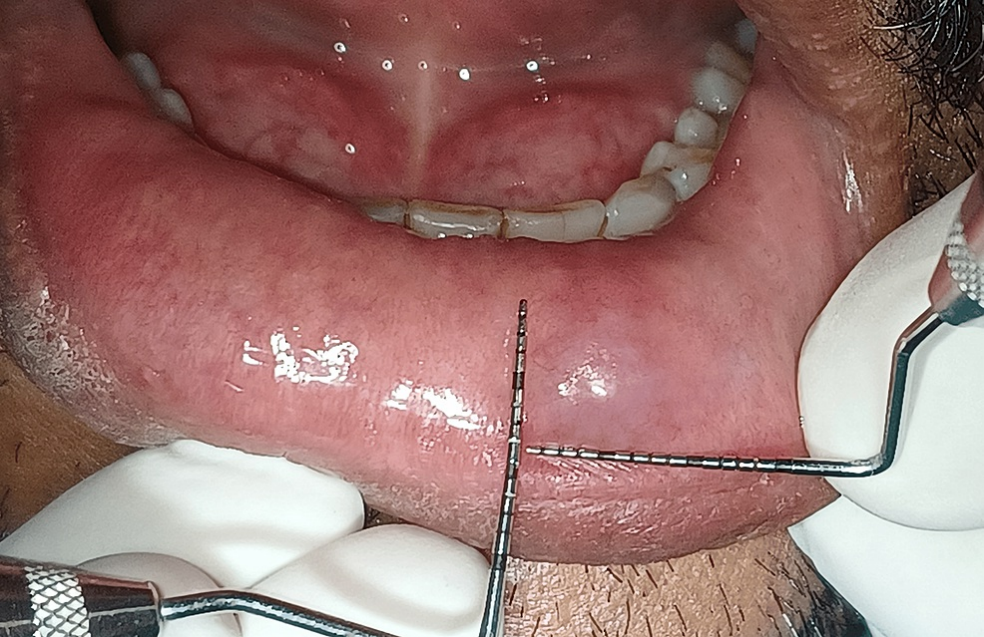
Measurements were taken of the lesion associated with lower lip.

On examination, a single, small, sessile, well-defined, ovoid, light blue-coloured swelling was present on the lower lip on the right side. Initially the swelling was smaller in size and gradually increased to its size to 8x8mm (Figure 2).

**Figure 3:**
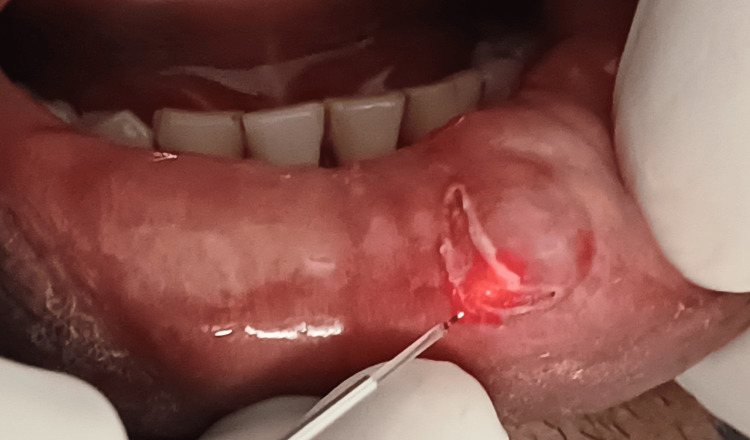
Surgical excision of the mucocele by diode laser.

**Figure 4:**
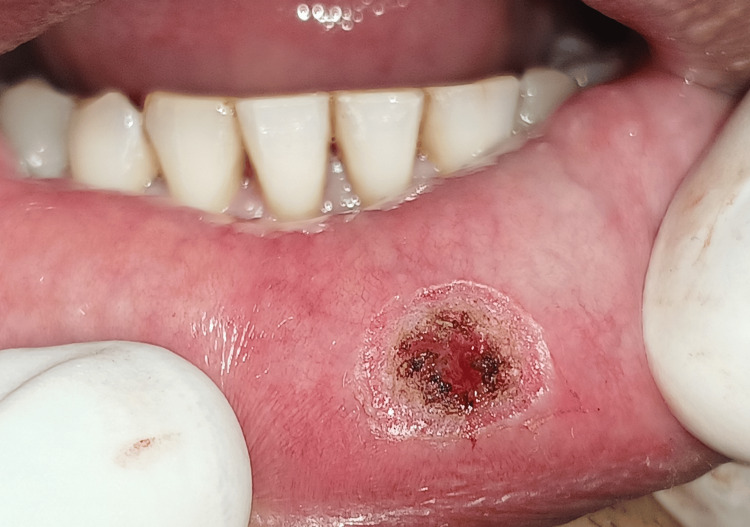
Complete surgical excision of the mucocele by diode laser done.

Patient gave no history of discharge from the swelling. On palpation the swelling was non-tender, soft and flocculent in consistency, on applying pressure the swelling depressed and on relieving the pressure the swelling reverted back. Teeth in the vicinity were not sharp or proclinated. Overjet and overbite were normal. After confirmation of inspector and palpatory findings, mucocele was considered under the provisional diagnosis. Among the clinical differential diagnoses were traumatic fibroma, lipoma, and fibrous hyperplasia. Patient was then advised for surgical treatment that involved excision by diode laser. The patient was referred for hematological examinations which were within the normal limits. The borders of the lesion were marked following all the protocols of the asepsis and sterilization and laser-assisted surgery was performed and total removal of the lesion was done with a diode laser (Biolase, Foothill Ranch, CA, USA) at 880nm, 10W by resecting it from the base to reduce chances of re-occurrence (Figure 3 and Figure 4).

**Figure 5:**
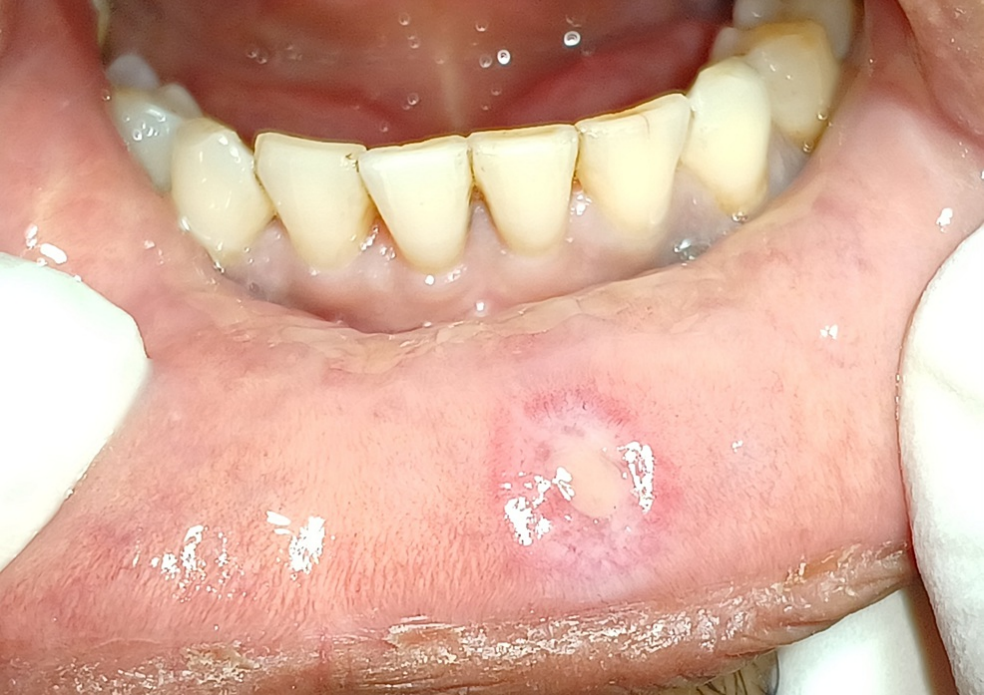
Postoperative view of the mucocele in healing phase after seventh day of surgery.

Post-operative instructions were given followed by medications and periodic recall. The patient was prescribed antiseptic gargles for a week and a nonsteroidal anti-inflammatory drug for three to four days. A softer diet was advised for the first two to three days. The patient was reviewed after the seventh day when the lesion was in a healing state as shown in Figure 5. 

**Figure 6:**
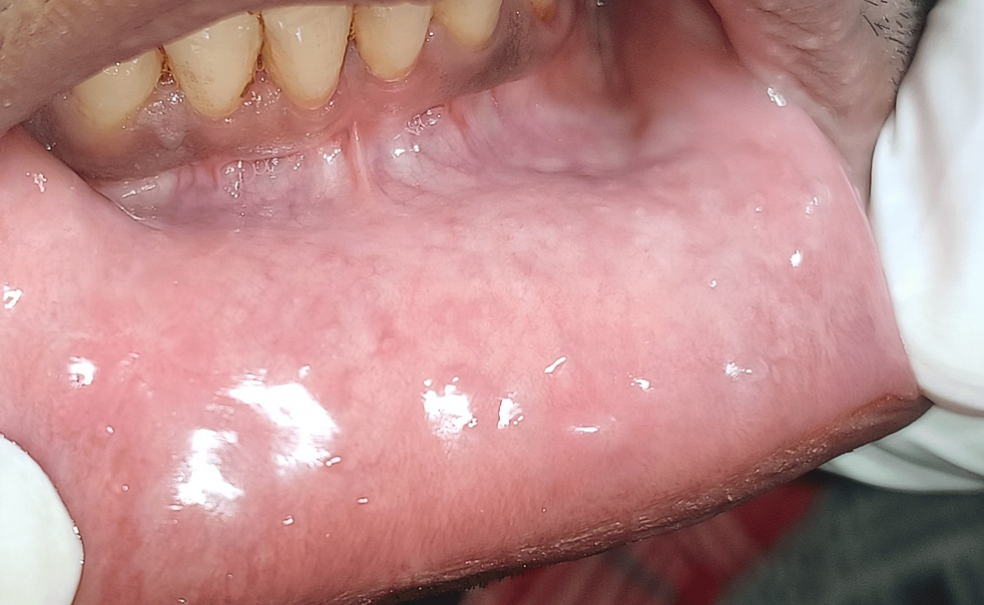
Recall examination after three months showed complete satisfactory healing and no recurrence.

Periodic examination after seven days revealed healing of the lesion that was satisfactory, while the next three months showed no recurrence with complete recovery (Figure 6).

**Figure 7:**
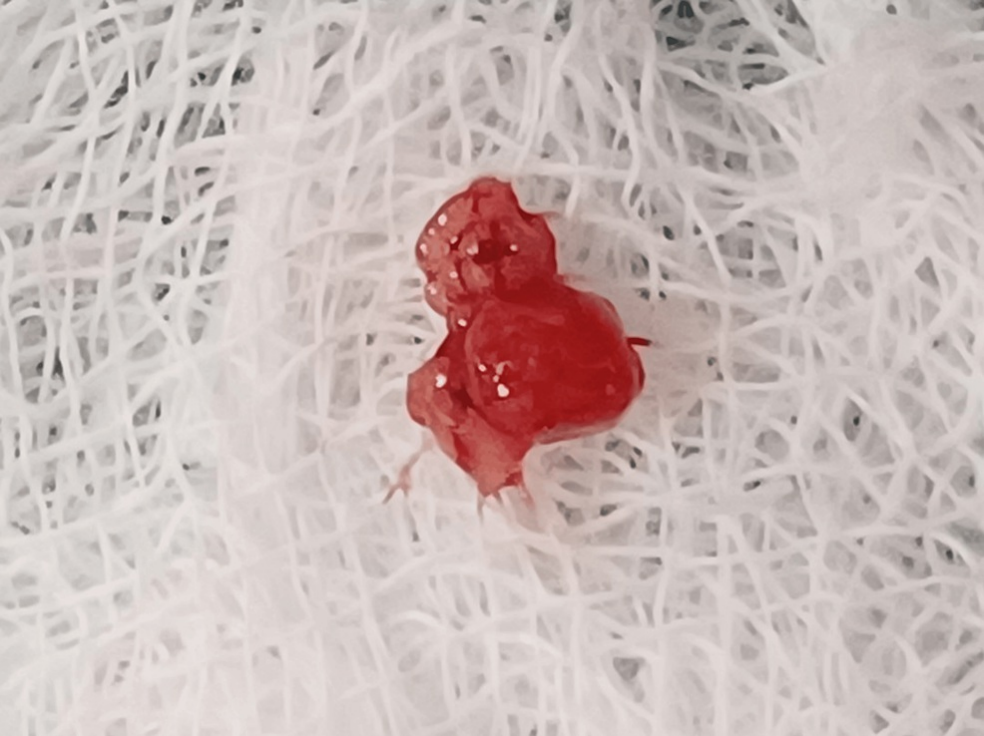
Specimen collected from the site by diode laser

**Figure 8:**
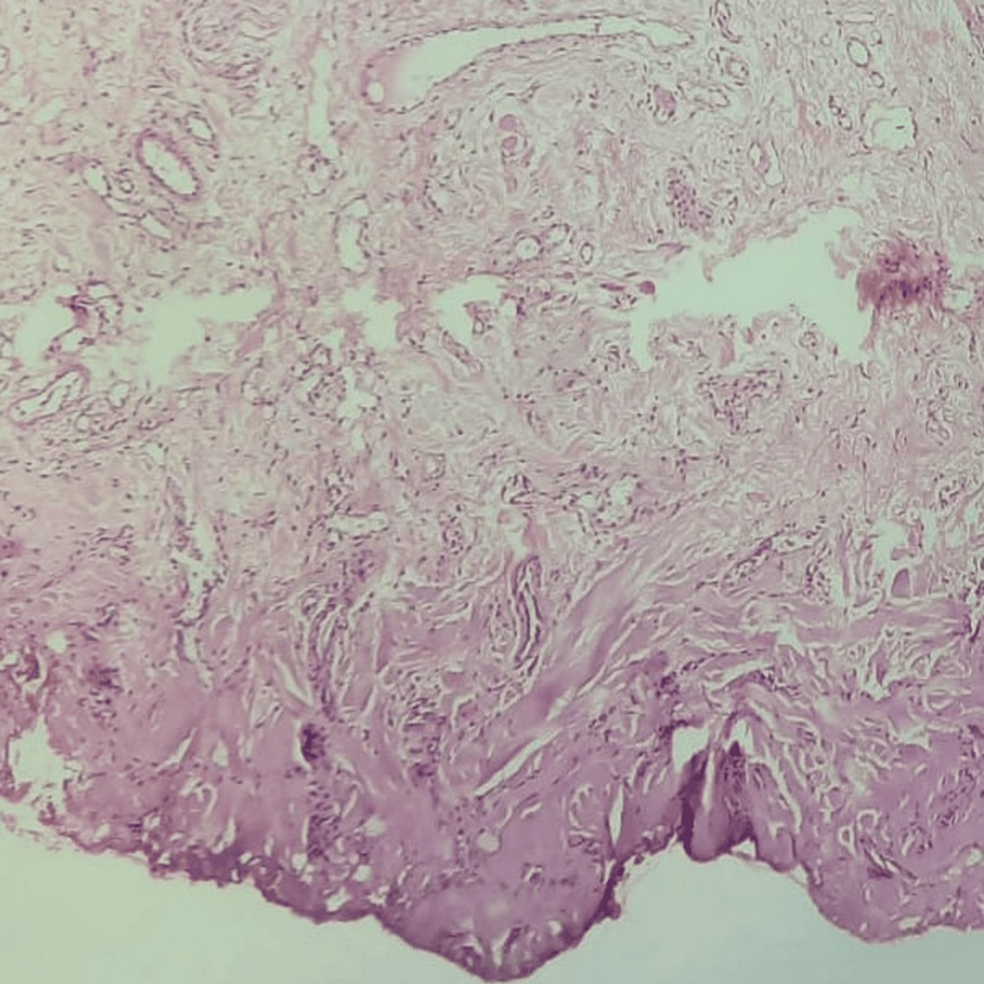
Histological image showing mucus collection patterns of mucus lesions with scarce amounts of connective tissue fibrosis.

The specimen was collected (Figure 7) and sent for histopathological examination, which confirmed the final diagnosis of mucous extravasation type of mucocele showing mucus collection to patterns of mature lesions with scarce amounts of mucus and connective tissue fibrosis (Figure 8). 

## Discussion

Non-neoplastic in nature, mucocele is a lesion of the salivary glands. Their occurrence bounds no age but is seen commonly in children and young adults with the peak incidence between 10-29 years. The most affected site anatomically is lower lip mucosa (67.4%) and other sites include the floor of the mouth, ventral tongue, and buccal mucosa [11,12]. The rupture of the excretory duct of the salivary gland, promoted by trauma, causes mucous extravasation to the surrounding tissue followed by inflammation. This is also termed mucous extravasation phenomenon [12,13]. Its occurrence on the lower lip is not apparent, their propensity to trauma is the most accepted hypothesis. The present case of mucocele occupied the lower lip secondary to lip biting. Depending on the tissue covering the lesion, this lesion typically has a soft, elastic consistency [14].

This lesion's histopathologic features ranged from patterns of mature lesions with sparse mucus and connective tissue fibrosis to acute inflammation blending with the mucus accumulation. Hyperplastic parakeratinized stratified squamous epithelium, mucus-filled cells in small cystic spaces, patches of spilled mucin encircled by granulation tissue, and sebaceous cells in the connective tissue can all be seen in the lesion [5].

The management however can be tailored to patient requirements. On the other hand, patient needs can be catered for in the management. Surgical excision is the most popular approach to treating this condition. Cryosurgery, electrocautery, marsupialization, intralesional corticosteroid injection, and CO2 laser ablation are other therapy possibilities [15-17]. The specific characteristics of the lesion and the patient requirements can decide the type of management referring to surgical or non-surgical [14]. In the above case, the surgical excision can ensure complete removal and less chances of recurrence. Recurrence can be decreased by removing the glandular acini that surround the lesion, excising or dissecting it down to the muscle layer, and preventing injury to the gland and duct that are nearby. The field of stomatology has been transformed after the induction of the laser. Laser-assisted surgeries offer a minimally invasive and precice approach resulting in reduced bleeding, enhanced healing, and increased patient comfort [12-17].

## Conclusions

Oral mucoceles are one of the most common lesions seen in children and young adults. Traditionally the surgical excision included marsupialization which was more invasive. The laser-assisted surgical methods are precise, less invasive, and patient-friendly, which are gaining popularity as they have minimal post-operative patient discomfort. Patients must be kept on periodic follow-ups to prevent the chances of recurrence.
